# Brain oscillations differentially encode noxious stimulus intensity and pain intensity

**DOI:** 10.1016/j.neuroimage.2017.01.011

**Published:** 2017-03-01

**Authors:** Moritz M. Nickel, Elisabeth S. May, Laura Tiemann, Paul Schmidt, Martina Postorino, Son Ta Dinh, Joachim Gross, Markus Ploner

**Affiliations:** a Department of Neurology and TUM-Neuroimaging Center, Technische Universität München, 81675 Munich, Germany; b Institute of Neuroscience and Psychology, University of Glasgow, Glasgow, UK

**Keywords:** Pain, Nociception, Oscillations, Tonic, Gamma, Alpha, Beta

## Abstract

Noxious stimuli induce physiological processes which commonly translate into pain. However, under certain conditions, pain intensity can substantially dissociate from stimulus intensity, e.g. during longer-lasting pain in chronic pain syndromes. How stimulus intensity and pain intensity are differentially represented in the human brain is, however, not yet fully understood. We therefore used electroencephalography (EEG) to investigate the cerebral representation of noxious stimulus intensity and pain intensity during 10 min of painful heat stimulation in 39 healthy human participants. Time courses of objective stimulus intensity and subjective pain ratings indicated a dissociation of both measures. EEG data showed that stimulus intensity was encoded by decreases of neuronal oscillations at alpha and beta frequencies in sensorimotor areas. In contrast, pain intensity was encoded by gamma oscillations in the medial prefrontal cortex. Contrasting right versus left hand stimulation revealed that the encoding of stimulus intensity in contralateral sensorimotor areas depended on the stimulation side. In contrast, a conjunction analysis of right and left hand stimulation revealed that the encoding of pain in the medial prefrontal cortex was independent of the side of stimulation. Thus, the translation of noxious stimulus intensity into pain is associated with a change from a spatially specific representation of stimulus intensity by alpha and beta oscillations in sensorimotor areas to a spatially independent representation of pain by gamma oscillations in brain areas related to cognitive and affective-motivational processes. These findings extend the understanding of the brain mechanisms of nociception and pain and their dissociations during longer-lasting pain as a key symptom of chronic pain syndromes.

## Highlights

• Stimulus intensity is encoded by alpha/beta oscillations in sensorimotor areas.
• Pain intensity is encoded by gamma oscillations in the medial prefrontal cortex.
• The encoding of stimulus intensity depends on stimulation side.
• The encoding of pain is independent of stimulation side.

## Introduction

Noxious stimuli induce physiological processes which commonly translate into the perception of pain (Adair et al., 1968, Price, 1999, Stevens, 1957). However, the translation of noxious stimuli into pain can vary substantially (Baliki and Apkarian, 2015). In particular, in chronic pain, the relationship between pain and noxious stimuli is often loose (Baliki and Apkarian, 2015). Such dissociations, however, occur not only in chronic pain but can also be observed in healthy human participants during a few minutes of experimental painful stimulation (Schulz et al., 2015), which offers the opportunity to gain experimental insights into the differential representation of noxious stimulus intensity and pain intensity in the human brain.

In the brain, noxious stimuli activate an extended network of brain areas including somatosensory, insular, cingulate and prefrontal cortices as well as subcortical and brainstem areas (Apkarian et al., 2005, Tracey and Mantyh, 2007). The activity of many of these brain areas correlates with both stimulus intensity and pain intensity (Coghill et al., 1999, Derbyshire et al., 1997, Loggia et al., 2012, Porro et al., 1998). Moreover, neurophysiological recordings disclosed that these brain areas yield neuronal responses at different frequencies ranging from theta (4–7 Hz) via alpha (8–13 Hz) and beta (14–29 Hz) to gamma (30–100 Hz) frequencies (Gross et al., 2007, Hauck et al., 2007, Mouraux et al., 2003, Ploner et al., 2006, Zhang et al., 2012). The amplitudes of these responses also co-vary with stimulus intensity and pain intensity (Gross et al., 2007, Schulz et al., 2011, Tiemann et al., 2015, Timmermann et al., 2001, Zhang et al., 2012). However, how these brain areas and brain responses differentially relate to stimulus intensity and pain intensity is not fully clear yet. Comparatively few studies explicitly distinguished between brain responses related to noxious stimulus intensity and pain. Although the results were not fully consistent, they showed that somatosensory cortices were more closely related to stimulus intensity whereas insular, cingulate and prefrontal cortices and their subdivisions were related to both stimulus intensity and pain intensity (Atlas et al., 2014, Baliki et al., 2009, Bornhovd et al., 2002, Buchel et al., 2002, Kong et al., 2006, Moulton et al., 2012). In addition, neurophysiological studies demonstrated that under some (Gross et al., 2007, Zhang et al., 2012) but not all (Tiemann et al., 2015) conditions, neuronal oscillations at gamma frequencies are more closely related to pain than responses at other frequencies. Most recently, we showed that a substantial dissociation of stimulus intensity and pain intensity can already be observed during 10 min of tonic painful heat stimulation (Schulz et al., 2015). Stimulus intensity was encoded by beta oscillations over sensorimotor cortex whereas pain intensity was encoded by gamma oscillations over the medial prefrontal cortex. However, the spatial specificity of the encoding of stimulus intensity and pain intensity, i.e. whether the representations of stimulus intensity and pain intensity depend on the location of the stimulus, has remained unclear.

To investigate the spatial specificity of the encoding of stimulus intensity and pain intensity, we applied painful tonic heat stimuli to the right and left hand of 39 healthy human participants. Concurrently, the participants provided continuous pain ratings and brain activity was recorded using electroencephalography (EEG). The results of linear mixed model analyses in source space show that stimulus intensity is stimulus location-dependently encoded by alpha and beta oscillations in sensorimotor areas contralateral to the stimulated hand whereas pain is encoded by gamma oscillations in the medial prefrontal cortex independent of stimulus location.

## Materials and methods

### Subjects

51 healthy human participants (age 24.7±5.6 years (mean±standard deviation), 24 female) participated in the experiment. All subjects were right-handed and gave written informed consent. Due to technical issues with the stimulation device, we had to exclude data sets of 12 subjects from further analysis. Thus, 39 participants (age 24.3±5.6 years, 18 female) were included in the final analysis. Participants were screened for depression (Beck Depression Inventory II (Beck et al., 1996), 5.3±4.3) and trait anxiety (State-Trait-Anxiety Inventory (Spielberger et al., 1983), female 33.6±3.9, male 39.0±8.0) to ensure that these traits were in the range of healthy subjects. Interviews confirmed that they did not suffer from neurological or psychiatric disorders or chronic pain and that they did not take any medication including analgesic drugs. The study was approved by the ethics committee of the Medical Faculty of the Technische Universität München and conducted in conformity with the declaration of Helsinki.

### Paradigm

The subjects participated in two *tonic pain* conditions and two *visual control* conditions. In the two *tonic pain* conditions, painful heat stimuli with a duration of 10 min were applied to the dorsum of the left (*tonic pain left*) or the right hand (*tonic pain right*), respectively. Apart from the side of stimulation, the two tonic pain conditions were identical. In both conditions, the subjects were instructed to continuously rate the perceived pain intensity on a visual analogue scale (VAS) ranging from 0 to 100 anchored at *no pain* and *worst tolerable pain* using a custom-built finger-span device with the non-stimulated hand. The scale was simultaneously presented on a screen by a vertical red bar, the length of which represented the current pain intensity rating.

**Fig. 1:**
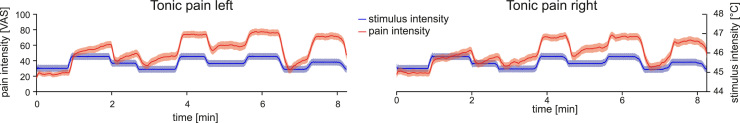
Time courses of stimulus intensity and pain intensity. Group mean time courses of stimulus intensity and pain intensity during *tonic pain left* and *tonic pain right* conditions. The blue and red shaded areas depict the standard error of the mean. VAS, visual analogue scale. (For interpretation of the references to color in this figure legend, the reader is referred to the web version of this article).

Painful heat stimuli were applied using a thermode (TSA-II, Medoc, Ramat Yishai, Israel). The time course of stimulation was similar for all subjects but the stimulus intensities were individually adjusted. Stimulus intensity was varied between three temperature levels (low, medium, high) of 0.5, 0.8 or 1.1 °C above an individually defined pain threshold temperature (see below). Thus, the stimulation continuously elicited sensations above pain threshold. In contrast to our previous study (Schulz et al., 2015) in which stimulus intensity was continuously adapted depending on the pain rating, the time course of stimulation was a priori defined in the present study. The three levels were applied using a sequence of 9 plateaus (Fig. 1) with 3 plateaus at each intensity. At each stimulus intensity, one plateau with a duration of 40, 50 and 60 s each was applied. The order of plateaus was pseudorandomized with the constraints that consecutive plateaus had differing stimulus intensities and that the sequence consisted of three consecutive triplets of low, medium and high stimulus intensities. The stimulation started at a baseline temperature of 40 °C, changes of stimulus intensity were implemented with a rate of 0.1 °C/s. Since stimulus intensities were individually adjusted, the time from the start of stimulation until the first plateau slightly varied between subjects. After the last plateau, the stimulus intensity decreased to the low intensity and stayed constant until the 10 min elapsed. The interval between the start of the first plateau and the end of the last decrease of stimulus intensity was included in the analysis resulting in an 8.2 min time window for analysis. Before the first tonic pain condition, individual pain threshold temperatures were determined. Over the course of 3 min, subjects were asked to adapt the stimulus intensity to their individual pain threshold using two keys of a keyboard to change the stimulus intensity with a rate of 0.5 °C/s. The pain threshold was defined as the average stimulus intensity during the last 10 s. The hand for which the threshold was determined was counterbalanced across subjects and the same threshold was then used to determine stimulation intensities for both hands.

To control for the sensory, motor and attentional components of the continuous pain rating procedure, we performed two *visual control* conditions (Baliki et al., 2006, Hashmi et al., 2013). In these two conditions, the temporally inverted time courses of the individual *tonic pain left* and *tonic pain right* ratings were visually presented as changes of the length of the vertical red bar over time. Subjects were instructed to continuously follow the length of the red bar using the finger-span device controlled by the right and the left hand, respectively. In both conditions, the thermode remained attached to the other hand at a neutral stimulus intensity of 32 °C.

The order of the *tonic pain left* and *tonic pain right* conditions was counterbalanced across subjects. The *tonic pain* conditions always preceded the respective *visual control* conditions. Stimulus presentation and timing was controlled using Matlab (Mathworks, Natick, MA, USA) and the Psychophysics Toolbox (http://psychtoolbox.org/).

### Recordings

During all conditions, EEG data were recorded using an electrode montage of 64 electrodes consisting of all 10–20 system electrodes and the additional electrodes Fpz, CPz, POz, Oz, Iz, AF3/4, F5/6, FC1/2/3/4/5/6, FT7/8/9/10, C1/2/5/6, CP1/2/3/4/5/6, TP7/8/9/10, P5/6 and PO1/2/9/10, plus 2 electrodes below the outer canthus of each eye (Easycap, Herrsching, Germany) and BrainAmp MR plus amplifiers (Brain Products, Munich, Germany). All electrodes were referenced to FCz and grounded at AFz. The EEG was sampled at 1000 Hz (0.1 μV resolution) and band-pass filtered between 0.016 Hz and 250 Hz. Impedances were kept below 20 kΩ. Continuous pain ratings and stimulus intensities were fed into the EEG system and recorded with the same sampling frequency.

### Preprocessing

Preprocessing was performed using BrainVision Analyzer software (Brain Products, Munich, Germany). EEG data were downsampled to 512 Hz, high-pass filtered at 0.5 Hz and 50 Hz line noise was removed using a regression approach from the BioSig software library (Vidaurre et al., 2011). Eye movements and muscle artifacts were corrected using independent component analysis (Jung et al., 2000) and all electrodes were re-referenced to the average reference. Subsequently, time intervals of 400 ms around data points exceeding ±80 μV and signal jumps exceeding ±30 μV were marked for rejection. Additionally, remaining artifacts were identified by visual inspection and rejected after the band-pass filtering for the time-frequency-analysis to avoid filter artifacts. No significant differences in percentage of rejected data were found (*tonic pain left/right*, 2.4±2.4%, 1.7±1.7%, *visual control left/right*, 1.9±1.7%, 2.2±1.8%, one-way repeated measures ANOVA, F_(3, 38)_=1.9, p=0.14).

### Time-frequency analysis

EEG data analyses were performed using the FieldTrip toolbox (Oostenveld et al., 2011) and custom programming in Matlab. First, the EEG data were band-pass filtered in theta (4–7 Hz), alpha (8–13 Hz), beta (14–29 Hz) and gamma (30–100 Hz) frequency bands using a fourth-order Butterworth filter (forward and backward). Second, time series of frequency-specific brain activity were computed in source space (see next section). Third, the Hilbert transform was applied and absolute values of the Hilbert transform, i.e. the amplitude within the respective frequency band, were computed. To decrease the amount of data for statistical analysis, we downsampled and smoothed the amplitude values of each frequency band as well as the time courses of stimulus intensity and pain ratings by using a moving average with a window length of 1 s and a step size of 0.1 s.

### Source analysis

We used linearly constrained minimum variance (LCMV) beamforming (Van Veen et al., 1997) to project the band-pass filtered data for each condition, frequency band and participant from electrode space into source space. Spatial filters were computed based on the covariance matrices of the band-pass filtered data for each frequency band and a lead field matrix. A three-dimensional grid with a 1 cm resolution covering the brain was defined. The lead field was constructed for each voxel using a realistically shaped three-shell boundary-element volume conduction model based on the template Montreal Neurological Institute (MNI) brain. We used a regularization parameter of 5% of the covariance matrix and chose the dipole orientation of most variance using singular value decomposition. Finally, the preprocessed EEG data were projected through the spatial filter to extract the amplitude time series of neuronal activity of each frequency band at each voxel.

### Statistical analysis

We performed all statistical analyses with the software environment R (R Core Team, 2016) and the *lme4* (Bates et al., 2015) package. We first compared pain ratings of the *tonic pain left* and *tonic pain right* conditions averaged across time by using a two-tailed paired t-test. Within subjects, pain ratings of both conditions showed a high correspondence (see Supplementary material for more details, Fig. S1).

We were next interested in the relationships of stimulus intensity and pain intensity with amplitude time courses in different frequency bands. To this end, we fitted linear mixed models (LMM) to the data of the *tonic pain left* and *tonic pain right* conditions. In contrast to our previous study (Schulz et al., 2015), main analyses were performed in source space. Stimulus and pain intensity were dependent variables and brain activity the independent variable for each voxel and each frequency band. The dependent and independent variables were z-transformed across all subjects and grouped for subjects. We included random intercepts and random slopes in the models to control for the between subject variability in average pain intensity and average stimulus intensity (random intercepts) and variability in slopes (random slopes). The slope of the fixed effects was used for statistical testing. This analysis, thus, assesses within subject effects but not between subjects effects. Moreover, it controls for between subjects differences in stimulus intensity and pain intensity. To control for multiple comparisons across all voxels, we applied the false discovery rate (FDR) correction (Benjamini and Hochberg, 1995). The final statistical t-maps were rendered to the template MNI brain and thresholded at p<0.05 (FDR corrected).

We performed three control analyses. First, to control for effects due to autocorrelation of the data, we fitted LMM with temporally inverted stimulus/pain intensity time courses. Second, to control for sensitization/habituation effects, we fitted LMM with linearly detrended pain ratings. Third, to control for the rating procedure and visual input, we fitted LMM to the data of the *visual control* conditions and performed conjunction analyses (see below) testing for significant relationships for both the left and the right *visual control* conditions. It is important to note that this analysis relates the length of the finger span but not the movement of the fingers to brain activity. The analysis can, thus, detect brain processes encoding the momentary length of the finger span and/or the visual bar but not brain processes encoding the movement of the fingers.

We next assessed the spatial specificity of the encoding of stimulus intensity and pain intensity, i.e. whether the observed effects depended on stimulus location. To this end, we first determined differences between *tonic pain left* and *tonic pain right* conditions using a contrast analysis. We specifically contrasted the two conditions by including condition (*tonic pain left* vs. *tonic pain right*) together with the interaction condition×brain activity as independent variables in the LMM. In addition to a whole-brain analysis, we performed a region of interest (ROI)-analysis using the bilateral primary somatosensory and motor cortices which have previously been implicated in the encoding of stimulus intensity (Gross et al., 2007, Schulz et al., 2015). ROIs were defined using Automated Anatomical Labeling (Tzourio-Mazoyer et al., 2002) and FDR correction was performed.

To determine similarities of the encoding of stimulus intensity and pain intensity between *tonic pain left* and *tonic pain right* conditions, we computed a conjunction analysis (Nichols et al., 2005) of the two conditions, identifying brain regions showing similar relationships of brain activity with stimulus or pain intensity for *tonic pain left* and *tonic pain right*. For those brain regions and frequency bands showing a significant conjunction for both stimulus and pain intensity, we further determined if brain activity in that brain region and frequency band was more closely related to stimulus intensity or pain intensity. We re-calculated the relationship between stimulus intensity and brain activity controlling for pain intensity and vice versa using LMM with brain activity as dependent variable and both stimulus intensity and pain intensity as independent variables. Again, a conjunction between *tonic pain left* and *tonic pain right* was computed which was termed controlled conjunction.

## Results

Mean pain threshold temperature was 44.7±1.1 °C (mean±standard deviation) resulting in an average stimulus intensity of 45.5±1.1 °C. Fig. 1 shows the group mean time courses of stimulus intensity and pain intensity for the *tonic pain left* and *tonic pain right* conditions within the 8.2 min time window of analysis. Average pain ratings of the *tonic pain left* and *tonic pain right* conditions did not differ significantly (53.3±23.0 and 49.6±23.4; t_(38)_=1.8, p=0.08). Within subjects, the pain ratings of the *tonic pain left* and *tonic pain right* conditions were remarkably similar (see Supplementary material for details, Fig. S1).

**Fig. 2:**
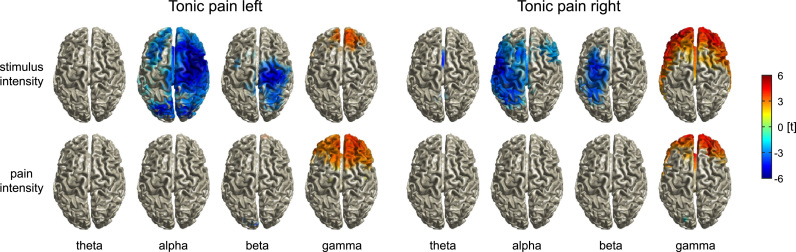
Brain oscillations encoding stimulus intensity and pain intensity. Linear mixed model-based whole-brain t-maps of the fixed effects showing the encoding of stimulus and pain intensity for different frequency bands. T-maps were thresholded at p<0.05, false discovery rate corrected for the whole brain.

We next investigated the encoding of stimulus intensity and pain intensity. We fitted linear mixed models (LMM) to the data of the *tonic pain left* and *tonic pain right* conditions in source space, resulting in whole-brain t-maps quantifying the strength of relationships of stimulus intensity and pain intensity with brain activity in different frequency bands (Fig. 2). The analyses revealed that stimulus intensity was encoded by alpha and beta activity in the sensorimotor cortex contralateral to the stimulated hand. Increasing stimulus intensity was associated with decreasing amplitudes of alpha and beta oscillations (alpha_tonic pain left_, t=−6.9, p<0.001, MNI: 40 10 60; alpha_tonic pain right_, t=−5.8, p<0.001, MNI: −50 −10 40; beta_tonic pain left_, t=−5.1, p<0.001, MNI: 20 −30 70; beta_tonic pain right_, t=−4.7, p<0.001, MNI: −30 −10 70 (MNI-coordinates in mm)). Stimulus intensity was further encoded by gamma activity in the prefrontal cortex (*tonic pain left*, t=4.2, p<0.001, MNI: 0 40 30; *tonic pain right*, t=6.5, p<0.001, MNI: −30 60 −10). In contrast, for both the left and the right hand, pain intensity was encoded by prefrontal gamma oscillations only (*tonic pain left*, t=4.1, p<0.001, MNI: 30 50 30; *tonic pain right*, t=4.8, p<0.001, MNI: −10 60 0), with stronger gamma oscillations indicating higher pain ratings. These results in source space were well compatible with findings in electrode space (Fig. S2).

Control analyses showed that the results cannot be explained by the autocorrelation of the data, sensitization/habituation effects or the rating procedure and the visual input. First, the significant relationships between brain activity and stimulus and pain intensity observed in the main analysis were not observed for the temporally inverted time courses. Second, analyses with linearly detrended pain intensity ratings yielded results similar to the main analyses. Detrended pain intensity was encoded by prefrontal gamma oscillations in the *tonic pain left* and *tonic pain right* conditions (*tonic pain left*, t=5.0, p<0.001, MNI: 0 40 30*; tonic pain right*, t=5.5, p<0.001, MNI: 10 50 40) and no significant relationships were observed at other frequencies. Third, in the *visual control* conditions, no significant conjunction of *visual controlleft* and *visual control right* for the relationship between brain activity in any frequency band and ratings was found.

**Fig. 3:**
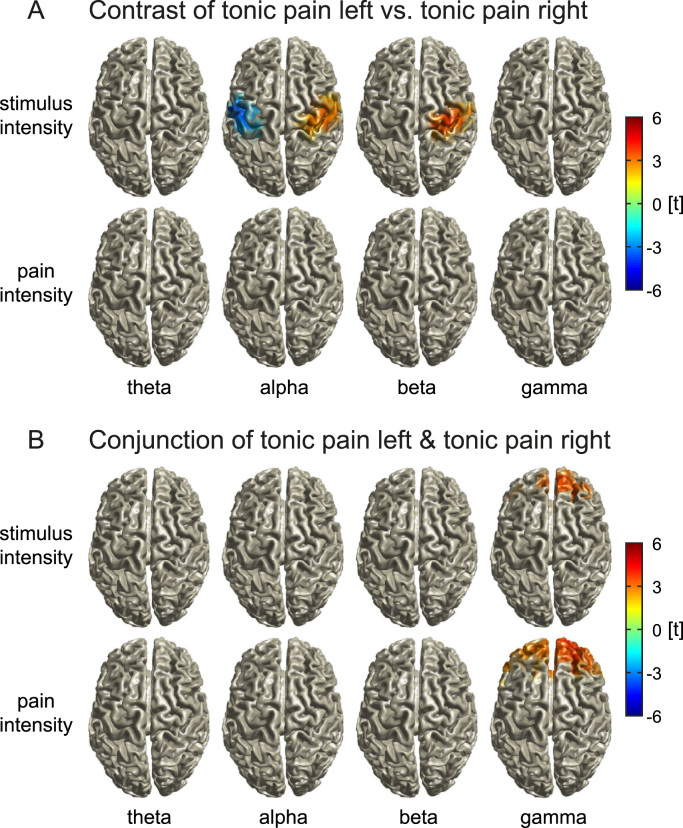
Differences and similarities between *tonic pain left* and *tonic pain right* in the encoding of stimulus intensity and pain intensity. A) T-maps showing significant differences between *tonic pain left* and *tonic pain right* in the encoding of pain intensity and stimulus intensity for different frequency bands. T-maps were masked for sensorimotor cortices and thresholded at p<0.05, false discovery rate corrected for the sensorimotor cortices. B) Whole-brain t-maps showing significant similarities (conjunction analysis) between *tonic pain left* and *tonic pain right* in the encoding of stimulus intensity and pain intensity for different frequency bands. T-maps were thresholded at p<0.05, false discovery rate corrected for the whole brain.

We next assessed the spatial specificity of the encoding of nociception and pain. To this end, we contrasted the *tonic pain left* and *tonic pain right* conditions. The whole-brain analysis did not show significant differences between the encoding of stimulus intensity or pain intensity during *tonic pain left* and *tonic pain right* conditions after FDR correction. A more sensitive ROI-analysis revealed significant differences in the left and right primary sensorimotor cortices for stimulus intensity only (alpha, t=3.6, p<0.001, t=−4.1, p<0.001; beta, t=3.8, p<0.001; Fig. 3A). No significant contrasts between *tonic pain left* and *tonic pain right* were found for the encoding of pain intensity. Thus, stimulus intensity but not pain intensity was stimulus location-dependently encoded by alpha and beta activity in the contralateral primary sensorimotor cortices.

**Fig. 4:**
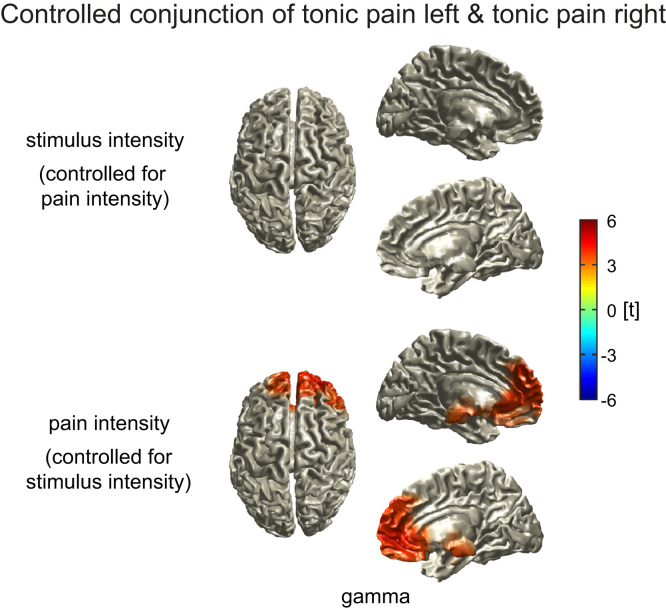
Similarities between *tonic pain left* and *tonic pain right* in the encoding of stimulus intensity controlled for pain intensity and vice versa. Whole-brain t-maps for the gamma frequency band showing significant similarities between *tonic pain left* and *tonic pain right* in the encoding of stimulus intensity when controlled for pain intensity (upper panel) and in the encoding of pain intensity when controlled for stimulus intensity (lower panel). T-maps were thresholded at p<0.05, false discovery rate corrected for the whole brain. The controlled conjunction analysis indicates that prefrontal gamma activity was more closely related to pain intensity than to stimulus intensity.

We further determined similarities between *tonic pain left* and *tonic pain right* conditions using a conjunction analysis (Fig. 3B). For the encoding of both stimulus and pain intensity, we observed conjunctions of *tonic pain left* and *tonic pain right* in the medial prefrontal cortex in the gamma band (stimulus intensity, t=3.8, p<0.001, MNI: 0 40 30; pain intensity, t=4.1, p<0.001, MNI: 30 50 30). However, the controlled conjunction analysis revealed that gamma activity in the medial prefrontal cortex was more closely related to pain intensity than to stimulus intensity (t=4.2, p<0.001, MNI: 20 70 10; Fig. 4). Thus, prefrontal gamma oscillations predominantly encode pain intensity independent of stimulus location. Finally, to investigate the translation of noxious stimuli into pain, we analyzed the connectivity between sensorimotor cortex and the medial prefrontal cortex (see Supplementary material for details). However, we did not find significant differences between *tonic pain* and *visual control* conditions.

## Discussion

In the present study, we investigated how stimulus intensity and pain intensity as measures of nociception and pain are differentially encoded in the human brain. To assess the spatial specificity of the encoding of stimulus intensity and pain, i.e. whether their representations depend on stimulus location, we applied tonic painful heat stimuli to the right and left hand of healthy human participants. Our findings show that stimulus intensity is negatively related to alpha and beta oscillations in sensorimotor areas contralateral to stimulus location. In contrast, pain is encoded by gamma oscillations in the medial prefrontal cortex independent of stimulus location. Thus, the translation from a noxious stimulus into pain is associated with a change from spatially specific encoding in sensory systems to spatially independent encoding in brain areas related to cognitive and affective-motivational brain systems.

The present observation of changes of alpha, beta and gamma oscillations during longer-lasting pain is in agreement with previous studies which found suppressions of alpha and beta oscillations (Giehl et al., 2014, Gram et al., 2015, Huishi Zhang et al., 2016, Nir et al., 2012, Peng et al., 2014, Shao et al., 2012) and increases of gamma oscillations (Dowman et al., 2008, Peng et al., 2014, Veerasarn and Stohler, 1992) during tonic pain. However, only a single previous study distinguished between brain processes related to stimulus intensity and pain intensity (Schulz et al., 2015). The present observation of the encoding of stimulus intensity and pain by alpha/beta and gamma oscillations, respectively, confirms the results of this previous study. Moreover, our results extend the previous study by revealing that the translation from a noxious stimulus into pain is associated with a change from a spatially specific encoding mode by alpha and beta oscillations to a spatially independent encoding mode by gamma oscillations. The encoding of a noxious stimulus is, thus, shaped by its sensory features and its location. In contrast, the encoding of tonic pain by gamma oscillations appears to be determined by spatially less dependent processes such as the salience, valence and/or motivational aspects of a noxious stimulus rather than by its precise sensory features.

How can the present findings on the encoding of stimulus intensity and pain be integrated with recent concepts of the functional significance of neuronal oscillations? A general concept assumes that gamma oscillations are related to the local encoding of information (Donner and Siegel, 2011). Our finding that pain is encoded by prefrontal gamma oscillations is well compatible with this concept and suggests that the encoding of longer-lasting pain is more closely related to emotional-motivational than to sensory processes (Hashmi et al., 2013, Schulz et al., 2015). Other recent concepts propose that gamma oscillations subserve feedforward influences (Bastos et al., 2015, Fries, 2015, Michalareas et al., 2016). Considering this framework, our findings might indicate that pain-related gamma oscillations in the medial prefrontal cortex impact on other brain regions, which eventually fulfill the biological function of pain, i.e. a behavioral response. Beyond, in predictive coding frameworks of brain function (Clark, 2013, Huang and Rao, 2011), gamma oscillations have been proposed to encode prediction errors (Arnal and Giraud, 2012, Bastos et al., 2012). As pain essentially signals the failure of predictions to protect the body, a close relationship between gamma oscillations and pain would be well compatible with this concept. With respect to oscillations at alpha and beta frequencies, a relation to long-range interactions has been proposed (Donner and Siegel, 2011) which regulate the excitability of functional brain systems (Jensen et al., 2014, Klimesch, 2012). Moreover, recent concepts relate alpha and beta oscillations to feedback signaling (Bastos et al., 2015, Fries, 2015, Michalareas et al., 2016) and, in a predictive coding framework, to the encoding of predictions (Arnal and Giraud, 2012, Bastos et al., 2012). Our findings might fit into these frameworks by showing that changes in nociceptive input are associated with changes of alpha and beta oscillations that might signal the feedback mediated adjustment of predictions.

The present results show how stimulus intensity as a proxy of nociception and pain are differentially represented in the human brain. The distinction between nociception and pain is central to understand the brain mechanisms of pain in health and disease. Under controlled experimental conditions, noxious stimuli commonly translate into the perception of pain (Adair et al., 1968, Price, 1999, Stevens, 1957). However, in everyday life, this translation process varies to flexibly adjust it to the current behavioral demands. Under most conditions, this variability is highly adaptive. In contrast, in chronic pain, when longer-lasting pain often occurs without adequate noxious stimuli and/or at abnormal low stimulus intensities (Baliki and Apkarian, 2015), the dissociation between nociception and pain is maladaptive. In such chronic pain states, the assumption of a linear translation of nociception into pain might result in an inappropriate focus on nociceptive processes and, in medical settings, in an improper diagnostic workup and insufficient therapy. Understanding the distinction between nociception and pain and the underlying brain mechanisms might therefore further the understanding, diagnosis and treatment of chronic pain.

The present paradigm and results provide novel experimental insights into the mechanism underlying dissociations between nociception and pain in healthy controls. These findings represent an important basis for understanding altered pain thresholds and dissociations of nociception and pain in chronic pain states. The dissociations observed in the present study are unlikely to be exclusively due to peripheral and/or spinal mechanisms. Modulations occurring at these levels would be more likely to affect stimulus intensity and pain intensity rather than dissociating the two measures. However, the precise mechanisms underlying the encoding of pain by prefrontal gamma oscillations and dissociations of stimulus intensity and pain intensity remain to be clarified. The integration of experimental and clinical evidence might help to further specify these mechanisms in health and disease.

In summary, our results reveal that during tonic painful stimulation stimulus intensity is spatially specifically encoded by alpha and beta oscillations in the contralateral sensorimotor cortex. In contrast, pain is encoded by gamma oscillations in the medial prefrontal cortex regardless of stimulation side. Thus, the translation of a noxious stimulus into pain is associated with a change from a spatially specific to a spatially independent encoding mode. These findings extend the understanding of the translation process of nociception into pain and its abnormalities which contribute to the pathology of chronic pain.
