# Supplementary material for: Brain oscillations differentially encode noxious stimulus intensity and pain intensity
Source: Neuroimage. 2017 Mar 1;148:141–7. doi: 10.1016/j.neuroimage.2017.01.011 (PMC5349759; doi:10.1016/j.neuroimage.2017.01.011)
Supplement: Supplementary file 1 — Supplementary material [file mmc1.pdf]

Supplementary Material for
Brain oscillations differentially encode noxious stimulus intensity and pain intensity

Moritz M. Nickel[1], Elisabeth S. May[1], Laura Tiemann[1], Paul Schmidt[1], Martina Postorino[1], Son Ta Dinh[1], Joachim Gross[2], Markus Ploner[1]

[1]Department of Neurology and TUM-Neuroimaging Center, Technische Universität München, 81675 Munich, Germany
[2]Institute of Neuroscience and Psychology, University of Glasgow, Glasgow, UK

Similarity of continuous pain ratings

We compared pain ratings of the *tonic pain left* and *tonic pain right* conditions averaged across time by using a two-tailed paired t-test. We further assessed the similarity of the pain ratings in the *tonic pain left* and *tonic pain right* conditions. We specifically tested whether the within-subject similarity of *tonic pain left* and *tonic pain right* ratings significantly exceeded the similarity between random pairs of *tonic pain left* and *tonic pain right* ratings. We therefore computed the Euclidean distance, i.e. the square root of the summed squared differences between corresponding time points of two time series. We specifically calculated the Euclidean distance for the z-transformed *tonic pain left* and *tonic pain right* ratings for each individual. We then compared the group mean Euclidean distance of these data to a permutation distribution of mean Euclidean distances of random pairs of *tonic pain left* and *tonic pain right* ratings retrieved from 5000 permutations. All values were z-transformed. Similarity was defined as the z-transformed distribution with flipped signs, so that increasing values indicate a higher similarity.

Within subjects, the pain ratings of the *tonic pain left* and *tonic pain right* conditions were remarkably similar (Fig. S1A). The within-subject similarity of *tonic pain left* and *tonic pain right* ratings significantly exceeded the similarity between random pairs of *tonic pain left* and *tonic pain right* ratings ($Z = 7.7$, $p < 0.001$; Fig. S1B). This similarity indicates that the translation from stimulus intensity to pain intensity, i.e. from nociception into pain, does not simply add random noise to the stimulus intensity time courses but represents an at least in part lawful and individually specific translation process. The rules and mechanisms of this translation process and its within-subject stability remain to be investigated. In the present study, the explicit or implicit memory of the first condition might have influenced the pain ratings of the second

condition and might thereby account for the observed within-subject similarity of pain ratings. However, it appears unlikely that a 10 minutes-time course of stimulation with changes of stimulus intensity every 40 to 60 seconds can be remembered after a single run. Future studies are needed to clarify this issue and to investigate whether the translation process of longer-lasting nociceptive stimulation into pain can be modeled analogous to shorter stimuli (Cecchi et al., 2012).

Connectivity analysis

To investigate the mechanisms that underlie the transition from stimulus intensity to pain, we examined the communication between cortical regions encoding stimulus intensity and pain intensity. To this end, we calculated undirected functional connectivity and directed (effective) connectivity between brain areas encoding stimulus intensity and pain intensity. To determine undirected functional connectivity, we calculated the debiased weighted phase lag index (dwPLI; Vinck et al., 2011) which is particularly robust against field spread, noise and sample-size bias. To determine directed connectivity, we calculated Granger causality (Granger, 1969).

Connectivity analyses were performed for three brain regions encoding stimulus intensity and pain intensity. For brain regions encoding stimulus intensity, the voxels with the strongest relationships between stimulus intensity and brain activity in the alpha/beta band in the *tonic pain left* and *tonic pain right* conditions were chosen. To this end, t-values of the LMM with alpha and beta activity as independent variables were summed and the voxels with the maximum negative t-values were identified. These voxels were located in the primary sensorimotor cortex, bilaterally (left, MNI: -40 -10 60; right, MNI: 40 -10 60 (MNI-coordinates in mm)). For brain regions encoding pain intensity, the voxel with the maximum t-value of the controlled conjunction between *tonic pain left* and *tonic pain right* using LMM with gamma activity as dependent variable and pain intensity as independent variable was determined (MNI: 20 70 10).

The raw time series of brain activity at the selected voxels were reconstructed using LCMV beamforming on EEG data without preceding band pass filtering, downsampling or smoothing. Data were segmented into epochs of 2 s with 50% overlap. To compute the dwPLI, the complex Fourier spectrum for each epoch was calcaluted using multitapers with ± 2 Hz frequency smoothing. The Granger causality spectra

were obtained by fitting autoregressive models on time-domain data (model order of 40) and subsequently using a spectral transfer function to get the frequency resolved spectrum. Granger causality as a measure of directed connectivity was computed from bilateral sensorimotor cortex to the medial prefrontal cortex. As the significance of connectivity values *per se* is undetermined, we compared connectivity values across conditions. Specifically, comparisons of connectivity spectra between *tonic pain* and *visual control* conditions were performed using cluster-based permutation statistics (Maris and Oostenveld, 2007) with 1000 permutations across conditions.

The comparisons of connectivity spectra between *tonic pain* and *visual control* conditions revealed no significant differences in the dwPLI spectra ($p > 0.1$ for both condition contrasts and region pairs) or in the Granger causality spectra ($p > 0.05$ for both condition contrasts and region pairs). We, thus, did not find patterns of undirected or directed functional connectivity serving the translation of a noxious stimulus into pain. However, this absence of significant effects neither rules out that brain areas encoding stimulus intensity and pain communicate with each other nor that this communication is implemented by neuronal synchrony. It may rather indicate that EEG and the applied connectivity measures are not sensitive to this communication process. Communication might be implemented via subcortical routes which are difficult to detect with EEG (Attal et al., 2012). Other approaches using intracerebral recordings, electrocorticography and magnetoencephalography and/or other connectivity measures or cross-frequency coupling might be more sensitive to these processes.

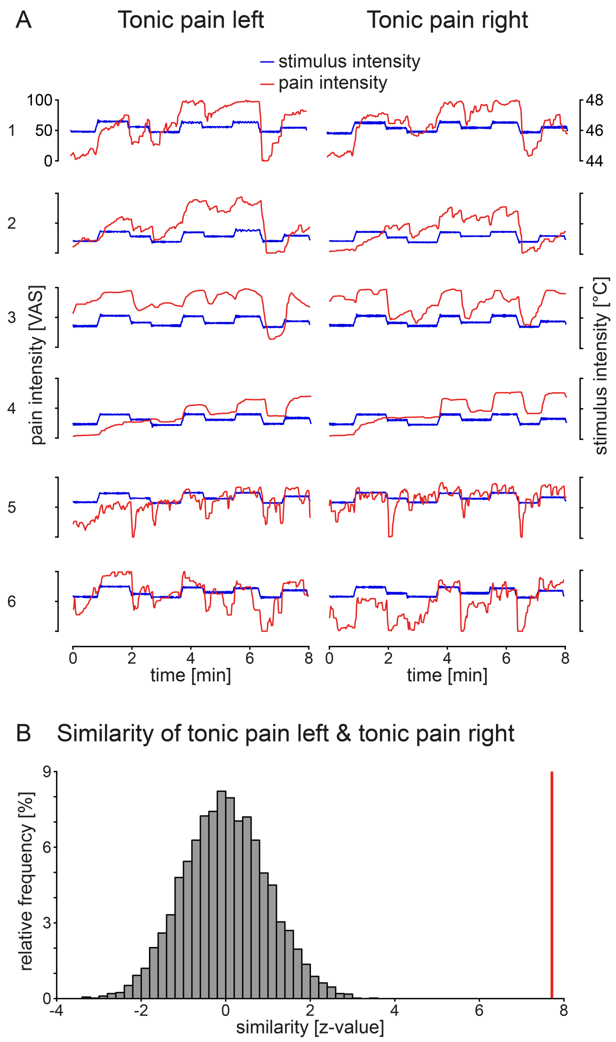

Figure S1. Similarity of pain ratings during *tonic pain left* and *tonic pain right* conditions. A, Examples. Individual time courses of stimulus intensity and pain intensity during *tonic pain left* and *tonic pain right* conditions of six subjects. B, Statistical analysis. The similarity between time courses of pain ratings was quantified using the z-transformed Euclidean distance. The group mean similarity of the individual's right and left hand pain ratings (vertical red bar) was compared to a permutation distribution with 5000 permutations. The within-subject similarity significantly exceeded the permutation distribution of similarity values ($Z = 7.7$, $p < 0.001$). VAS, visual analogue scale.

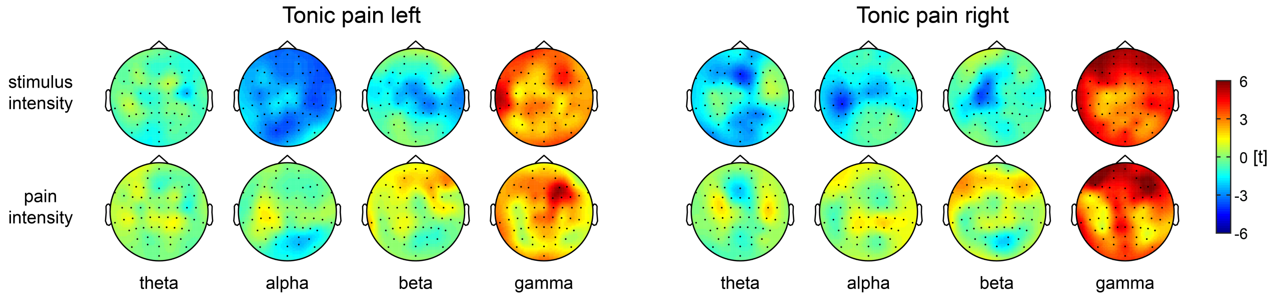

Figure S2. Brain oscillations encoding stimulus intensity and pain intensity. Linear mixed model-based t-topographies of the fixed effects showing the encoding of stimulus intensity and pain intensity for different frequency bands on electrode level. Please note that the topographies show unthresholded results.

References

Attal, Y., Maess, B., Friederici, A., David, O., 2012. Head models and dynamic causal modeling of subcortical activity using magnetoencephalographic/electroencephalographic data. Rev Neurosci 23, 85-95.

Cecchi, G.A., Huang, L., Hashmi, J.A., Baliki, M., Centeno, M.V., Rish, I., Apkarian, A.V., 2012. Predictive dynamics of human pain perception. PLoS Comput Biol 8, e1002719.

Granger, C.W.J., 1969. Investigating Causal Relations by Econometric Models and Cross-Spectral Methods. Econometrica 37, 414-&.

Maris, E., Oostenveld, R., 2007. Nonparametric statistical testing of EEG- and MEG-data. J Neurosci Methods 164, 177-190.

Vinck, M., Oostenveld, R., van Wingerden, M., Battaglia, F., Pennartz, C.M., 2011. An improved index of phase-synchronization for electrophysiological data in the presence of volume-conduction, noise and sample-size bias. Neuroimage 55, 1548-1565.
